# Coding-complete genome sequence of an isolate of papaya virus E in tomato

**DOI:** 10.1128/MRA.00344-23

**Published:** 2023-08-18

**Authors:** Peter Abrahamian, Dimitre Mollov, Rosemarie W. Hammond, Yazmin Rivera

**Affiliations:** 1 US Department of Agriculture, Animal and Plant Health Inspection Service, Plant Protection and Quarantine, Science and Technology, Plant Pathogen Confirmatory Diagnostic Laboratory, Laurel, Maryland, USA; 2 US Department of Agriculture, Agriculture Research Service, Horticultural Crops Disease and Pest Management Research Unit, Corvallis, Oregon, USA; 3 US Department of Agriculture, Agriculture Research Service, Beltsville Agricultural Research Center, Molecular Plant Pathology Laboratory, Beltsville, Maryland, USA; Katholieke Universiteit, Leuven, Belgium

**Keywords:** *Cytorhabdovirus*, tomato, emerging virus

## Abstract

An isolate of papaya virus E was identified in tomato fruits from Mexico. The coding-complete genome sequence was determined using high-throughput sequencing. The coding-complete genome is 13,412 nucleotides and contains 8 open reading frames.

## ANNOUNCEMENT

*Cytorhabdovirus caricae* [papaya virus E (PpVE); syn. bean-associated cytorhabdovirus; syn. citrus-associated rhabdovirus] belongs to the genus *Cytorhabdovirus* in the subfamily *Betarhabdovirinae* and family *Rhabdoviridae*. PpVE was found in papaya and bean (1, 2), weed species in the family *Fabaceae* (3), citrus, passion fruit, paper bush, and soybean (4, 5). PpVE occurs in South America (Brazil and Ecuador) and China and is transmitted by the whitefly (*Bemisia tabaci*) (3, 6). In this study, we report the coding-complete genome sequence of an isolate of PpVE from a tomato (*Solanum lycopersicum* L.). In December 2019, a tomato fruit sample showing marbling symptoms, typical of the tomato brown rugose fruit virus, was intercepted in fruit lots from Mexico.

The fruit sample was homogenized using the Fast Prep (MP Biomedicals, Irvine, CA), and total RNA was extracted using the Qiagen RNeasy Plant Mini Kit (Qiagen, Hilden, Germany). RNA and library quality were evaluated using a 4200 TapeStation System (Agilent Technologies, Santa Clara, CA) and quantified using a Qubit fluorometer (ThermoFisher Scientific, Waltham, MA). A total of 400 ng RNA was used for library preparation using the Illumina TruSeq Stranded Total RNA Kit with Ribo-Zero Plant (Illumina Inc., San Diego, CA) according to the manufacturer’s instructions. Libraries were denatured and diluted to 2.5 pM and loaded onto an Illumina NextSeq 550 (Illumina, Inc.) and sequenced in 75 bp single-end reads. Reads were trimmed using Trimmomatic v.0.39 (7) with a quality score above 30. Trimmed tomato reads were excluded by filtering reads against the tomato reference genome version SL3.0 (GCF_000188115.4) using bowtie v.1.3.1 (8); the remaining non-host reads were assembled using Metaviral SPAdes v. 3.15.02 (9) using default settings. Contigs were analyzed using BLAST+ against a locally curated NCBI viral database (https://www.ncbi.nlm.nih.gov/labs/virus/vssi/). The contig was annotated in Geneious Prime 2022.1.1 (geneious.com) and ORFfinder (ncbi.nlm.nih.gov/orffinder/). A sequence alignment of the annotated contig with other representative cytorhabdovirus genomes was obtained using MAFFT v7.490 (10). Maximum-likelihood trees were prepared using RaxML v.8.2.11 (11) with 500 bootstraps in Geneious Prime 2022.1.1.

**Fig 1:**
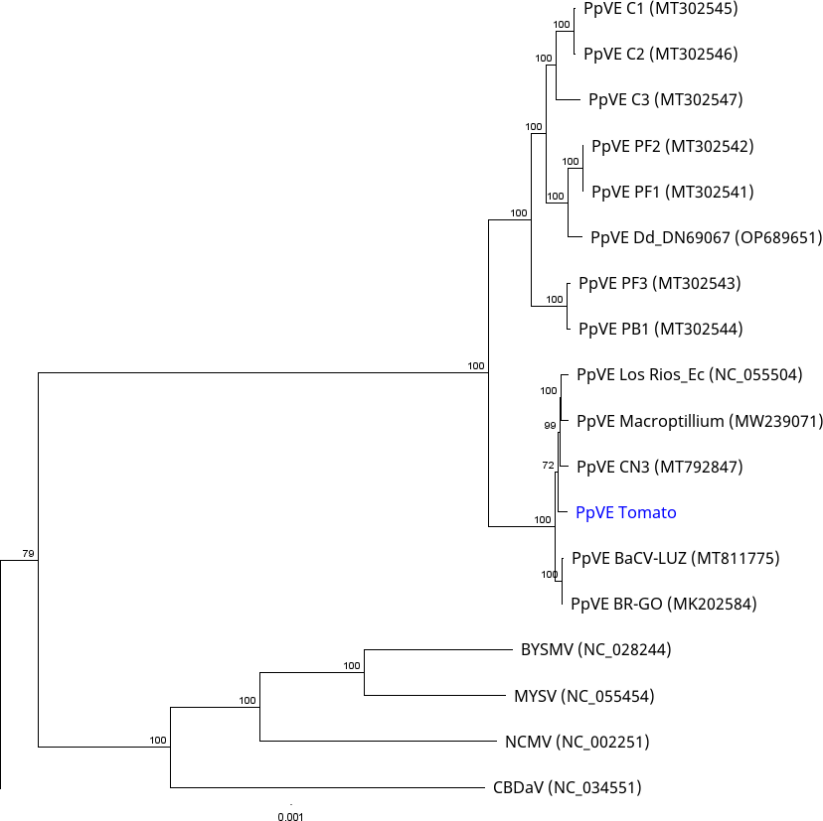
Maximum-likelihood tree based on the coding-complete genome of representative *Cytorhabdovirus* species. The isolate sequenced in this study is highlighted in blue. The tree was subject to 500 boostraps, and support values above 70% are indicated on the branches. Abbreviations: BYSMV, barley yellow striate mosaic virus; CBDaV, colocasia bobone-disease-associated virus; MYSV, maize yellow striate virus; NCMV, northern cereal mosaic virus; and PpVE, papaya virus E.

A total of 76,385,793 raw reads, out of which 16,150,256 were host reads, were obtained. Contig assembly produced a single contig of 13,412 bp with a GC content of 44.5%. Only 18,746 reads were mapped to the PpVE Los Rios reference isolate (NC_055504) with a ref-seq coverage of 99.6%. The 5′ and 3′ termini were incomplete and missing 20 bp and 39 bp, respectively, compared to the PpVE-Los Rios isolate. The virus, designated PpVE-tomato, has a total of eight open reading frames encoding the following genes, N, P, P3, P4, P5, M, G, and L (Table 1). The conserved sequence motif 3′-AUUCUUUUUG-5′ was found between all open reading frames (ORFs) except for P3/P4 and P4/P5. This motif was also found in the leader sequence with an additional U at the +2 site. The complete pairwise identity of the genome showed the highest similarity to PpVE BR-GO at 96.8%. Phylogenetic analysis placed this isolate clustering with other isolates of PpVE from South America but not from Asia (Fig. 1). The biological significance of this PpVE isolate on tomato is yet to be determined.

**TABLE 1:** Pairwise identity of papaya virus E with closely related representative isolates for the different open reading frames and coding-complete genome

		Nucleotide (a.a) %ID					
	Length [nt (a.a)]	PpVE BR-GO^*a*^	PpVE Los Rios_EC	PpVE Dd_DN69067	PpVE PF1	PpVE C1	PpVE PB1
*N*	1,356 (452)	97.6 (98.5)	96.7 (97.6)	83.5 (94.0)	83.6 (94.2)	82.7 (92.2)	83.8 (94.5)
*P*	1,338 (446)	95.8 (94.8)	96.5 (95.7)	77.8 (84.5)	77.6 (84.5)	77.9 (83.6)	78.8 (84.3)
*P3*	570 (190)	96.3 (97.4)	97.5 (97.4)	82.1 (91.0)	82.5 (88.9)	81.1 (91.0)	81.2 (90.5)
*P4*	237 (79)	97.5 (94.9)	–	68.3 (59.5)	69.2 (59.5)	–	67.9 (59.5)
*P5*	138 (46)	*–*^*b*^	92.0 (89.3)^*c*^	–	–	–	–
*M*	645 (215)	97.5 (99.5)	97.2 (99.1)	85.1 (96.3)	84.3 (97.2)	81.8 (90.7)	81.7 (95.8)
*G*	1,560 (520)	97.6 (99.0)	96.9 (97.7)	78.1 (87.9)	79.2 (87.1)	–	78.5 (86.7)
*L*	6,342 (2,114)	97.4 (98.5)	97.1 (98.0)	80.6 (93.3)	80.6 (93.8)	80.8 (92.6)	80.0 (93.2)
*Coding-complete^d^*	13,412	96.8	96.6	77.8	77.9	77.4	77.5

^*a*^ The papaya virus E (PpVE) isolates were reported from the following hosts: bean (PpVE BR-GO; MK202584), papaya (PpVE Los Rios_EC; NC_055504), soybean (PpVE Dd_DN69067; OP689651), citrus (PpVE C1; MT302545), passion fruit (PpVE PF1; MT302541), and paper bush (PpVE PB1; MT302544) (2, 4, 6).

^*b*^ “–” indicates missing open reading frame.

^*c*^ P5 in PpVE Los Rios_EC is 51 nucleotides shorter than in PpVE tomato.

^*d*^ The representative genomes were trimmed to the length of PpVE tomato.

## Data Availability

The complete annotated coding sequence of the PpVE tomato isolate was deposited in GenBank under the accession no. OQ418488. The raw read files were deposited in the sequence read archive (SRA) database under BioProject PRJNA961054.
